# M cell-dependent antigen uptake on follicle-associated epithelium for mucosal immune surveillance

**DOI:** 10.1186/s41232-018-0072-y

**Published:** 2018-09-03

**Authors:** Yutaka Nakamura, Shunsuke Kimura, Koji Hase

**Affiliations:** 1 Division of Biochemistry, Faculty of Pharmacy, Keio University, Tokyo, 105-0011 Japan; 2 Graduate School of Medicine, The University of Tokyo, Tokyo, 108-8639 Japan; 3 Laboratory of Histology and Cytology, Graduate School of Medicine, Hokkaido University, Sapporo, 060-8638 Japan; 4 International Research and Development Center for Mucosal Vaccines, The Institute of Medical Science, The University of Tokyo, Tokyo, 108-8639 Japan

**Keywords:** FAE, M cell, Mucosal immune surveillance, Antigen uptake, Mucosa-associated lymphoid tissue (MALT), Mucosal infection

## Abstract

The follicle-associated epithelium (FAE) covering mucosa-associated lymphoid tissue is distinct from the villous epithelium in cellular composition and functions. Interleukin-22 binding protein (IL-22BP), provided by dendritic cells at the sub-epithelial dome region, inhibits the IL-22-mediated secretion of antimicrobial peptides by the FAE. The Notch signal from stromal cells underneath the FAE diminishes goblet cell differentiation. These events dampen the mucosal barrier functions to allow luminal microorganisms to readily gain access to the luminal surface of the FAE. Furthermore, receptor activator of nucleic factor-kappa B ligand (RANKL) from a certain stromal cell type induces differentiation into microfold (M) cells that specialize in antigen uptake in the mucosa. Microfold (M) cells play a key role in mucosal immune surveillance by actively transporting external antigens from the gut lumen to the lymphoid follicle. The molecular basis of antigen uptake by M cells has been gradually identified in the last decade. For example, GPI-anchored molecules (e.g., glycoprotein 2 (GP2) and cellular prion protein (PrP^C^)) and β1-integrin facilitate the transport of specific types of xenobiotics. The antigen transport by M cells initiates antigen-specific mucosal immune responses represented by the induction of secretory immunoglobulin A (S-IgA). Meanwhile, several invasive pathogens exploit M cells as a portal to establish a systemic infection. Recent findings have uncovered the molecular machinery of differentiation and functions of M cells.

## Background

Epithelial cells lining on the body surface play essential roles in various tissue functions, including respiration, digestion, reproduction, and evacuation. The epithelium is continuously exposed to external materials, which entails the risks of encountering a lot of pathogens (e.g., bacteria, viruses, and parasites) and their toxins. Indeed, various kinds of pathogens invade a host body through mucosal epithelial layers to cause infectious diseases, such as salmonellosis, hemorrhagic colitis, shigellosis, tuberculosis, influenza, and acquired immunodeficiency syndrome (AIDS). Moreover, a multitude of microbes colonize mucosal tissue, particularly human intestine, which harbor approximately 40 trillion bacteria [1, 2]. To deal with such a microbial burden, a unique immune system, termed “the mucosal immune system,” has evolved in vertebrates. The mucosal immune system is unambiguously distinguished from the systemic immune system in terms of cellular composition, antigen recognition, and effector functions.

One of the most characteristic features of the mucosal immune system is inherent lymphoid tissue called the mucosa-associated lymphoid tissue (MALT). MALT consists of multiple or solitary lymphoid follicles covered by the follicle-associated epithelium (FAE) with a dome-like shape. These well-organized lymphoid structures are constitutively found in the intestinal and nasopharyngeal tracts and are inducible in the bronchial tissue [3]. The systemic lymphoid tissue, namely the spleen and peripheral lymph nodes, activates the immune response to antigens from the blood vessels and afferent vessels, respectively. Meanwhile, MALT directly takes up antigens from the lumen across the FAE to induce the mucosal immune response. Thus, the mucosal immune system continuously monitors exogenous antigens on the mucosal surface for immune surveillance.

The antigen internalization via the FAE was initially found by Kenzaburo Kumagai in 1922 [4]. However, the cellular entity responsible for the antigen transport had been unclear for half a century since the finding, mainly owing to technical difficulties. In the 1970s, by taking advantage of the development of electron microscopy, Max Cooper found the specialized epithelial cells that play a key role in the antigen uptake in the FAE in the bursa of Fabricius [5]. Concomitantly, Robert Owen revealed a similar cell type in human Peyer’s patches and named it microfold (M) cells [6, 7]. It is well known that antigen uptake through microfold (M) cells contributes to the induction of antigen-specific immunoglobulin A (IgA), a dominant isotype in secretory fluids of most mucosal tissues, except for the respiratory and genital tract [8]. IgA secreted into the mucosal lumen binds to luminal commensal and pathogenic microbes to prevent microbial adhesion to epithelial cells. Therefore, antigen uptake by M cells is considered to be critical for the onset of the mucosal immune response. Recent studies have uncovered the molecular basis of differentiation and functions of M cells. In this review, we discuss current knowledge of development and antigen uptake in the FAE and M cells.

## Formation of Peyer’s patches and the FAE

Formation of MALT, especially Peyer’s patches (PP), during developmental stages has been well documented. Anlagen of mouse PPs are found at 15 days postcoitus in mice [9]. In the primordial PPs, lymphotoxin α_1_β_2_ (LTα_1_β_2_)-expressing lymphoid tissue inducer (LTi) cells stimulate LTβR-expressing lymphoid tissue organizer (LTo) cells to produce chemokines (e.g., CXCL13) and adhesion molecules (e.g., VCAM-1) involved in the recruitment and organization of lymphocytes [10]. At embryonic day 17.5 (E17.5), the dome-shaped FAE is established, and it produces CCL20 to attract CCR6-expressing B cells and immature CD11b^+^ dendritic cells [11–13]. Transcriptome analysis illustrated a distinct gene expression profile between the FAE and the villous epithelium [14]. Remarkably, several chemokines (*Ccl6*, *Ccl9*, *Ccl20*, *Ccl26*, and *Cxcl16*) are highly upregulated in the FAE [15–17]. Expressions of these chemokines are mainly controlled by signaling through the LTβR and receptor activator of nucleic factor-kappa B (RANK; TNFRSF11A). Genetic ablation of RANK signaling diminishes *Ccl20* expression in gut-associated lymphoid tissue (GALT) [18]. In human, the clusters of lymphocytes are identified in small intestine at 14–16 weeks of gestation, and PPs are microscopically observable at gestational age of 24 weeks [19]. After birth, human PPs greatly expand early in life [19, 20].

The FAE in PP is formed at the late stage of fetal development as described above. We previously reported that LTo cell-mediated activation of epithelial Notch signaling contributes to the organization and integrity of the FAE [21]. Activation of epithelial Notch signaling suppresses goblet cell differentiation as described below and secures CCL20 expression in the FAE, facilitating full maturation of PPs and isolated lymphoid follicles. The maturation of MALT also necessitates antigen transport via M cells. In support of this idea, mice lacking M cells because of deficiency in RANK in intestinal epithelium or nucleic factor-kappa B ligand (RANKL) in sub-epithelial mesenchymal cells [known as M cell inducer (MCi)] of GALTs display the reduced size of PPs in association with inactivation of the germinal center reaction [18, 22]. Thus, FAE-intrinsic Notch signaling as well as antigen exposure is essential for the maturation of GALTs. Luminal antigens are also indispensable for the establishment of the overall mucosal immune system. Antigen-free mice that are raised and bred on an elemental diet, devoid of dietary antigens under germ-free conditions, exhibited a marked reduction of lymphocytes in the small intestinal lamina propria and mesenteric lymph nodes, but not in the spleen [23].

## Characterization of the FAE

**Fig. 1:**
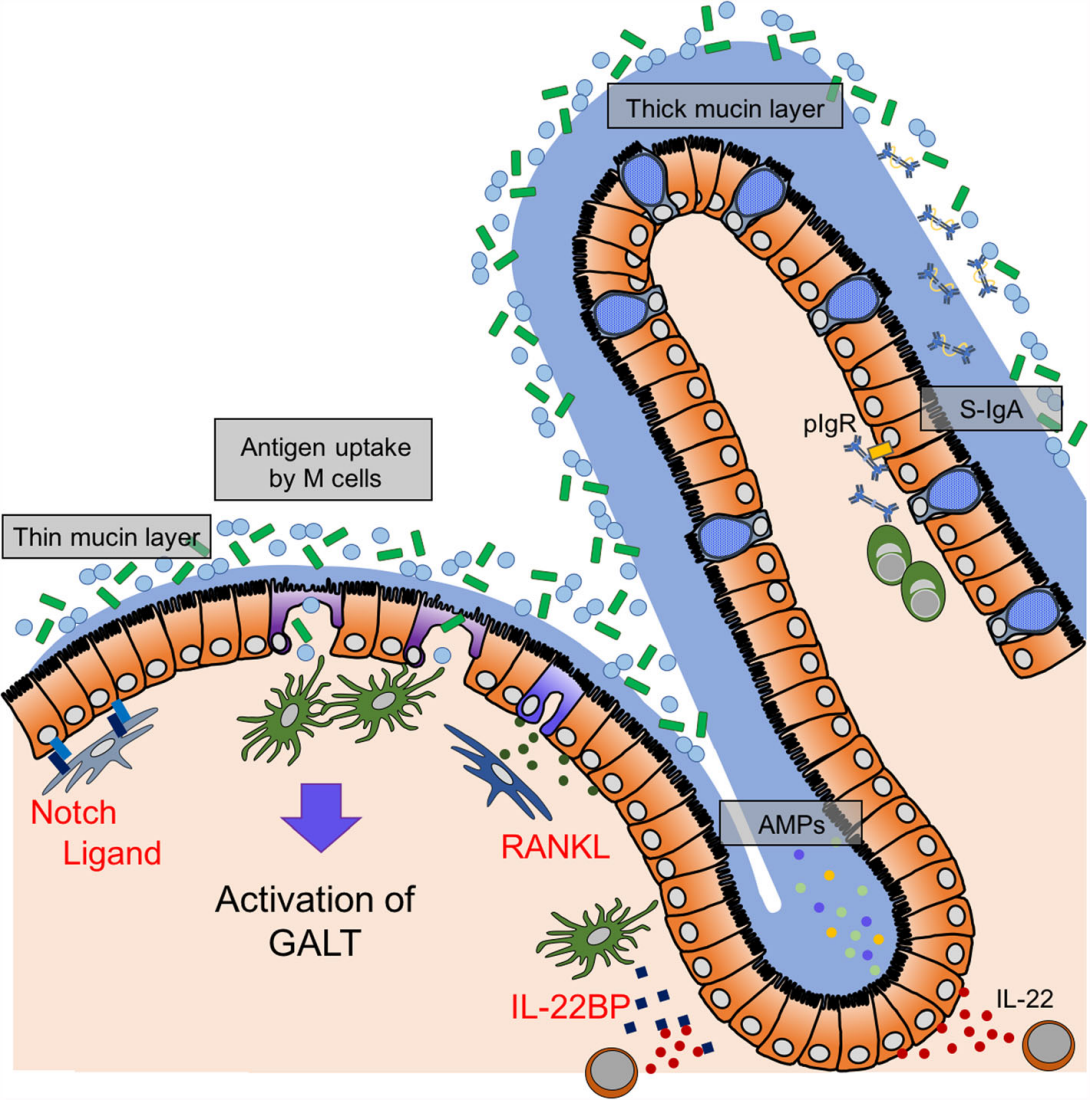
M cells in the FAE specialize in antigen uptake on the mucosal surface. To protect against bacterial invasion, the villous epithelium is equipped with robust mucosal barriers composed of tight junctions, thick mucin layer, S-IgA, and AMPs. In contrast, the FAE is vulnerable because of a thin mucin layer and downregulation of the expression of polymeric immunoglobulin receptor (pIgR) and AMPs. Consequently, external antigens are able to easily gain access to M cells on the FAE. The expression of the Notch ligand and IL-22BP in the sub-epithelial region, at least partially, account for the attenuated barrier functions

Intestinal epithelial cells constitute a front-line barrier for the prevention of invasive microorganisms. For instance, intercellular tight junctions provide a robust physical barrier by securing close connections between adjacent cells [24]. Polymeric immunoglobulin receptor (pIgR) expressed on the basolateral plasma membrane of epithelial cells transports dimeric IgA to the lumen [25]. Furthermore, Atoh1/Math1^+^ intestinal secretory cell lineages, such as goblet cells, play central roles in the establishment of physicochemical barriers by secreting mucin [26]. These molecules are a prerequisite for segregation of microbial habitats from the epithelial surface [27]. In sharp contrast to the ordinary villous epithelium, the FAE is mainly composed of enterocytes and M cells with a limited number of goblet cells. The mucin layer is therefore thinner in the FAE region than in the villous region [28]. The hypoplastic mucin layer allows luminal antigens to readily gain access to the FAE (Fig. 1).

The differentiation of goblet cells is controlled by the Notch signal that is widely utilized for cell-cell interaction in various cell types [26]. In the intestinal villi, secretory-type epithelial cells express Notch ligands (e.g., *Dll1*) to bind Notch receptors in adjacent epithelial cells [29, 30]. The ligation of the ligands liberates the Notch intracellular domain (NICD), which translocates into the nucleus to form a transcriptional activator complex with the recombination signal binding protein for immunoglobulin κ J region (RBP-J). The NICD/RBP-J complex upregulates the expression of *Hes1*, which in turn represses the expression of *Atoh1*, the main regulator of secretory cell lineages [26, 31, 32]. Consequently, activation of the Notch signal in intestinal epithelial cells limits the number of secretory cells and maintains the balance between absorptive and secretory epithelial cell populations. Such self-regulation of epithelial cell populations in the intestine is termed lateral inhibition. In PPs, stromal cells beneath the FAE constitutively express a Notch ligand, *Dll1* [21, 33], indicating that secretory cell lineages in the FAE are suppressed by stromal Notch ligands (Fig. 1). The inactivation of the Notch signal by genetic ablation of RBP-J in intestinal epithelial cells (RBP-J^ΔIEC^) markedly increases the number of goblet cells in both the FAE and villous epithelium [21]. As a consequence, RBP-J^ΔIEC^ mice are defective in maturation of PPs and isolated lymphoid follicles at least partly because of downregulated expression of CCL20, which is mainly produced by enterocytes, but not goblet cells, during the developmental stage.

In addition, Paneth cells abundantly produce antimicrobial products (AMPs) like lysozyme, RegIIIγ, and α-defensins (cryptdins) in response to cholinergic nerve activation and stimuli with microbial products [34, 35]. Enterocytes in the intestinal villi also produce AMPs, such as RegIIIγ and β-defensins [34, 36, 37]. However, the expression of the AMPs remarkably decreases in the FAE as compared with the villus epithelium. Interleukin-22 (IL-22), produced by type 3 innate lymphoid cells (ILC3) and T helper 17 (Th17) cells in the lamina propria, upregulates the expression of AMPs [38, 39]. IL-22 signaling is ameliorated in the FAE. This is attributed to constitutive expression of IL-22-binding protein (IL-22BP), a secreted decoy receptor for IL-22, which is abundantly provided by immature dendritic cells at the SED of PPs [40] (Fig. 1). Expression of pIgR is also downregulated in the FAE [41], although the underlying mechanism remains to be clarified. Collectively, the cellular components of PPs, namely epithelial cells, dendritic cells, and stromal cells, are responsible for the establishment of a specialized microenvironment that facilitates uptake of mucosal antigens.

## Differentiation of M cells

**Fig. 2:**
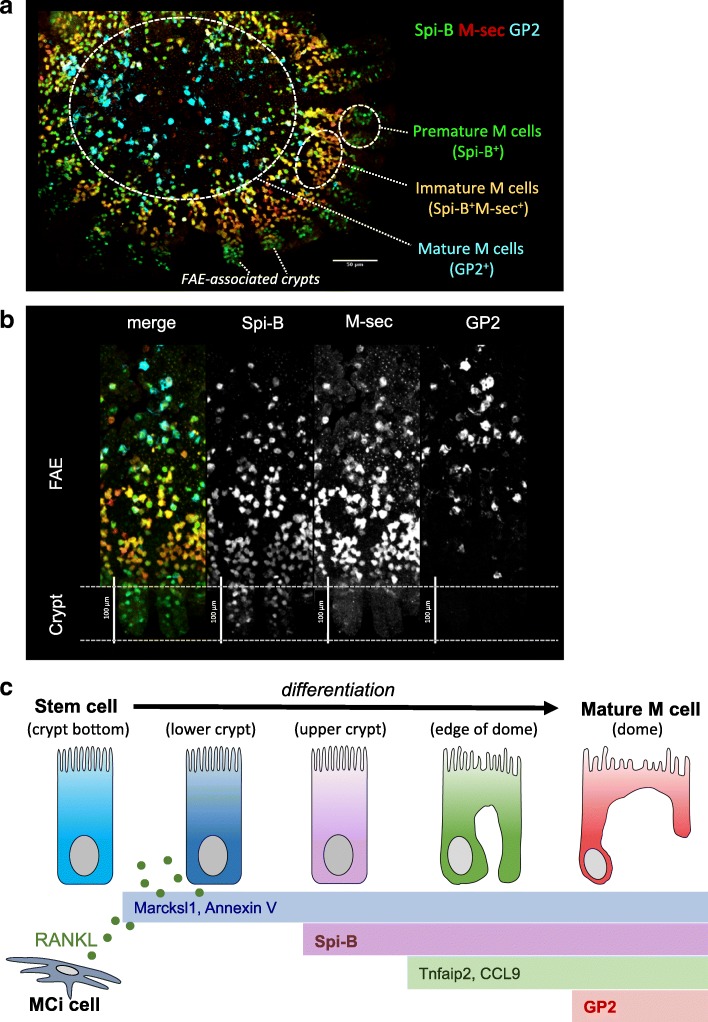
Expression of M cell marker molecules during differentiation. **a** Fluorescence in situ hybridization (FISH) image of *Spib*, *M-Sec*, and *Gp2* in a mouse FAE specimen is shown. Premature and immature M cells are observed at the crypt and the periphery of dome region, respectively, whereas fully mature M cells are found at the middle and upper dome region. **b** Enlarged images of the cypt region are shown. **c** RANKL from stromal M cell inducer (MCi) cells stimulates intestinal stem cells to initiate M cell differentiation. Early (Marksl1, AnnexinV), intermediate (Spi-B, Tnfaip2, CCL9), and mature (GP2) markers are serially expressed during the course of differentiation

M cells account for approximately 10% of FAE cells in mouse PPs (Fig. 2a) [28]. M cells can be identified by electron microscopy owing to their characteristic morphology: sparse and irregular microvilli, called microfold, as well as an invaginated basal plasma membrane to form the pocket-like structure that is occupied by immunocompetent cells [5, 6]. Although M cells were anatomically identified in the 1970s, technical difficulties in isolation and culture of M cells had hampered further analyses to elucidate their differentiation and functions. To deal with this issue, we previously established a method to isolate the FAE and successfully performed transcriptome analysis followed by in situ hybridization to determine M cell-specific molecules [14, 42]. Consequently, the course of analysis led to the identification of several M cell-specific markers including CCL9, Sgne-1, and GP2. Furthermore, recent progress uncovered the key molecules that govern the differentiation of M cells. Williams and colleagues revealed that RANKL (TNFSF11) is essential for the differentiation of M cells [43]. As described earlier, RANKL is provided from stromal MCi cells residing underneath the FAE of GALT (Fig. 2) [18]. Because intestinal epithelial cells constitutively express a RANKL receptor, RANK (TNFRSF11A), intraperitoneal administration of recombinant RANKL ectopically induces differentiation of the M cell-like GP2^+^ cells in the intestinal villous region [43, 44]. Of note, M cells scattered in the intestinal villi of RANKL-treated mice and the FAE of untreated mice, suggesting existence of potential machinery to regulate the number of M cells. Because we observed that deficiency in RBP-J did not increase the number of M cells (Hase K et al., unpublished observation), lateral inhibition via Notch signaling should be excluded in the regulation of M cell number.

The stimulation with RANKL activates NF-κB family in both canonical (NF-κB1 p50, RelA, and c-Rel) and non-canonical (NF-κB2 p52 and RelB) pathways [45]. The RANKL treatment in *aly*/*aly* mice carrying a point mutation of NF-κB inducing kinase (NIK) gene failed to induce M cells in the intestinal villi [46, 47]. Because NIK acts as a specific component of the non-canonical NF-κB pathway [48], this result suggested that the RANKL-induced non-canonical NF-κB pathway is essential for M cell differentiation. Recent studies reported that M cell differentiation was dependent on non-canonical RelB pathway, but not canonical c-Rel [46, 47, 49]. However, the canonical NF-κB pathway supports M cell differentiation by enhancing the expression of *Relb* and *Nfkb2* in enteroids stimulated with TNF-α [47].

Over the course of differentiation, M cells differentially express several molecular markers [50–52], suggesting that M cells undergo stepwise maturation processes (Fig. 2b). This concept was supported by a current single-cell transcriptome analysis that clearly demonstrated molecular signatures of early and late M cell progenitors, and immature and fully mature M cells [53].

Indeed, *Marcksl1* and *Anx5* are expressed in M cell progenitors and villus epithelial cells immediately after intraperitoneal treatment with RANKL [44, 53]. In contrast, Spi-B, Ccl9, and Tnfaip2 (also termed M-sec) were recognized as immature M cell makers [53]. Among these molecules, Spi-B plays a pivotal role in the development of fully differentiated M cells. Glycoprotein-2 (GP2)-positive mature M cells are absent in PPs of *Spib*^−/−^ mice, whereas Marcksl1^+^AnnexinV^+^ immature M cells are intact [44]. Furthermore, *Spib*^−/−^ mice reveal much less uptake of pathogenic bacteria, such as *Salmonella enterica* serovar Typhimurium (*Salmonella* Typhimurium) and *Yersinia enterocolitica* [44]. Collectively, the nuclear translocation of RelB and the expression of transcription factor Spi-B are essential for the RANKL-induced differentiation of M cells. In contrast, the commensal bacteria *Alcaligenes* can be internalized into GALT of *Spib*^−/−^ mice [54, 55], indicating that immature M cells may take up this PP-colonizing bacteria. Alternatively, the epithelial cell-independent trans-epithelial antigen sampling by mononuclear phagocytes may mediate the internalization of *Alcaligenes* in PPs [56]. This observation implies that antigen uptake of certain bacteria in the FAE does not always necessitate mature M cells.

## Antigen uptake receptors in M cells

**Fig. 3:**
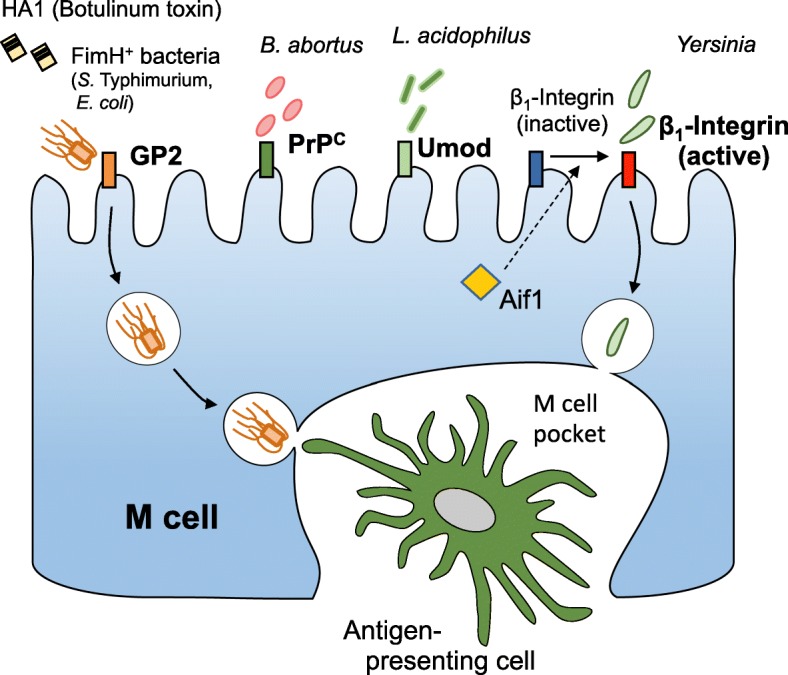
Antigen uptake receptors on the apical surface of M cells. GP2 binds to the HA1 domain of the botulinum toxin and FimH^+^ type 1 pili of certain bacterial species. PrP^C^ facilitates internalization of *Brucella abortus* into M cells. Umod serves as an uptake receptor for *Lactobacillus acidophilus*. β_1_-integrin is activated by Aif1 to function as an uptake receptor for *Yersinia* spp. Antigens taken up by M cells are transcytosed to an M cell pocket, whereby antigen-presenting cells, like dendritic cells, capture the antigens

Recent studies demonstrated that M cells utilize several receptors to recognize and transport specific luminal antigens. GP2 is a GPI-anchored protein expressed on the apical surface of M cells to function as an uptake receptor for type I pili-expressing bacteria (e.g., *S.* Typhimurium and *Escherichia coli*) (Fig. 3) [42]. GP2 also binds hemagglutinin A1 of botulinum neurotoxin, increasing susceptibility to botulism [57]. M cells highly express other GPI-anchored membrane proteins, cellular prion protein (PrP^C^) and uromodulin (Umod)/Tamm-Horsfall protein (THP), which serve as uptake receptors for *Brucella abortus* and *Lactobacillus acidophilus*, respectively [55, 58, 59]. In addition, β_1_-integrin localized on the apical surface of M cells facilitates transcytosis of *Yersinia* spp. [58, 60] (Fig. 3). These observations illustrate that M cells express multiple receptors on their apical plasma membrane to efficiently take up certain microbes. Although the uptake receptors have been extensively analyzed during this decade, the intracellular trafficking machinery regulating the antigen transcytosis remains largely unknown. A current study revealed that Allograft inflammation factor 1 (Aif1), which is known to be involved in phagocytosis in macrophages [61], is specifically upregulated in M cells among intestinal epithelial cells. Aif1 plays a non-redundant role in the activation of β_1_-integrin and facilitates the uptake of *Y. enterocolitica* [60]. Furthermore, transcriptome analysis of M cells demonstrated that M cells abundantly express a substantial number of intracellular molecules, potentially contributing to vesicular transport or actin remodeling during the course of the transcytotic pathway [53]. Functional analyses of these molecules should open up a new research direction on the molecular mechanism of M cell-specific antigen transport.

## M cell as a portal for infectious agents

Several invasive bacteria are known to exploit M cells to invade the host body. *S.* Typhimurium and *Shigella* spp. gain entry to Peyer’s patches through M cells [62–64]. Furthermore, M cells take up scrapie prion protein (PrP^Sc^) from food and thus potentially contribute to the accumulation of PrP^Sc^ in follicular dendritic cells of PPs [65]. The deletion of M cells moderates the pathogenesis of prion’s disease [66]. In addition to the foodborne pathogens, the airborne pathogen *Mycobacterium tuberculosis* also invades the host body via M cells in nasopharynx-associated lymphoid tissues (NALTs) and bronchus-associated lymphoid tissues (BALTs) [67]. These findings indicate that M cells function as an initial step of both mucosal immunity and pathogenesis, and this character is often referred to as the “double-edged sword” [68].

However, little is known about whether M cell-dependent antigen uptake predominantly contributes to immune responses against infection or bacterial invasion. Our current study reveals that M cell-null mice are highly susceptible to the mucosal infection of non-invasive pathogens, indicating that M cell-mediated antigen transport is important for the host defenses against, at least, non-invasive pathogens (Nakamura et al. unpublished observation). Recent studies have revealed that gastrointestinal microbiota is implicated in the development of multiple diseases, such as diabetes, obesity, multiple sclerosis, and autism spectrum disorder [69]. There is an open question whether M cell-mediated antigen transport and the subsequent immune response could regulate the commensal microbiota. Further investigations may uncover novel links among M cells, mucosal immunity, and the gut microbiota.

## Conclusion

Recent findings have provided new insights into the molecular basis of antigen transport on the mucosal surface. In particular, identification of receptors specific for pathogens and/or commensals on M cells exemplify selective uptake of particular antigens for mucosal immunosurveillance. Because M cell-mediated antigen uptake contributes to the induction of antigen-specific secretory immunoglobulin A (S-IgA), the uptake receptors on M cells may be a promising target for mucosal vaccination to efficiently induce pathogen-specific S-IgA [70–72]. S-IgA not only suppresses pathogenic infection but also shapes the gut microbial community. Alteration of the gut microbial composition is a predisposing factor for various diseases including diabetes, obesity, non-alcoholic steatohepatitis, multiple sclerosis, and autism spectrum disorder [69]. It is therefore intriguing to evaluate the role of M cells in the regulation of the gut microbiota, as well as the development of the diseases associated with dysbiosis. Further investigation employing M cell-null mice will shed light on the biological significance of M cells.

## Abbreviations

AIDS: Acquired immunodeficiency syndrome
Aif1: Allograft inflammation factor 1
AMPs: Antimicrobial proteins
BALT: Bronchus-associated lymphoid tissue
FAE: Follicle-associated epithelium
GALT: Gut-associated lymphoid tissue
GP2: Glycoprotein 2
IgA: Immunoglobulin A
IL-22: Interleukine-22
IL-22BP: Interleukine-22 binding protein
ILC3: Type 3 innate lymphoid cell
LTi: Lymphoid tissue inducer
LTo: Lymphoid tissue organizer
LTα_1_β_2_: Lymphotoxin α_1_β_2_
M cell: Microfold cell
MALT: Mucosa-associated lymphoid tissue
MCi cell: Microfold cell inducer cell
NALT: Nose-associated lymphoid tissue
NICD: Notch intracellular domain
NIK: NF-κB inducing kinase
pIgR: Polymeric immunoglobulin receptor
PP: Peyer’s patch
PrP^C^: Cellular prion protein
PrP^Sc^: Scrapie prion protein
RANK: Receptor activator of nucleic factor-kappa B
RANKL: Receptor activator of nucleic factor-kappa B ligand
RBP-J: Recombination signal binding protein for immunoglobulin κ J region
S-IgA: Secretory immunoglobulin A
Th17: T helper 17
THP: Tamm-Horsfall protein
Umod: Uromodulin
